# Effects of solar‐simulated (UVB plus UVA) radiation on the skin microbiome: An exploratory study

**DOI:** 10.1111/php.70078

**Published:** 2026-01-29

**Authors:** Florian Dimmers, Doreen Reichert, Claudia Wigmann, Carles Trullàs, Jaime Piquero‐Casals, Anthony Brown, Monica Foyaca, Charlotte Esser, Jean Krutmann

**Affiliations:** ^1^ IUF – Leibniz‐Research Institute for Environmental Medicine Duesseldorf Germany; ^2^ Department of Dermatology HELIOS Klinikum Krefeld, Academic Teaching Hospital of the University of Aachen Aachen Germany; ^3^ Innovation and Development, ISDIN Barcelona Spain; ^4^ Department of Dermatology Dermik Multidisciplinary Dermatology Clinic Barcelona Spain

**Keywords:** skin microbiome, flow cytometry, human skin, UVA, UVB, in vivo

## Abstract

This exploratory *in* *vivo* study investigated the impact of solar‐simulated ultraviolet (UV) radiation (UVB plus UVA) on the composition of the human skin microbiome in healthy male volunteers. Thirty Caucasian men were exposed to suberythemal and erythemal doses of UV radiation (0.5, 0.7, and 1.0 minimal erythema dose, MED) on defined areas of the lower back. Skin swabs were collected from both irradiated (*n* = 243) and nonirradiated control sites (*n* = 81) 30 min, 24 h, and 96 h postexposure. The microbial profiles were generated using flow cytometry, and the data were analyzed via the open‐access bioinformatic platform FlowSoFine™. The results revealed pronounced alterations in the microbial composition, with changes already detectable 30 min after exposure. Although partial recovery was observed over time, certain microbial shifts persisted. Further analysis indicated dose‐dependent trends in microbiome changes, suggesting a potential relationship between the extent of microbial alteration and the applied UV dose. These results suggest that even low, nonerythematous exposure to solar‐simulated UV radiation can rapidly alter the microbial balance of the skin and emphasize the role of UV radiation as a potent modulator of the skin microbial homeostasis.

## Graphical abstract

This exploratory *in* *vivo* study investigated the effects of solar‐simulated ultraviolet radiation (UVB plus UVA) on the human skin microbiome. Healthy male volunteers were exposed to suberythemal and erythemal UV doses, and skin microbiota were analyzed at multiple time points using flow cytometry‐based profiling and the open‐access bioinformatic platform FlowSoFine™. UV exposure induced rapid, dose‐dependant alterations in microbial community composition, detected as early as 30 min postirradiation and already at suberythemal dose levels. Partial recovery was observed over time, while certain changes persisted, indicating that everyday, nonerythematous UV exposure may influence skin microbial homeostasis.

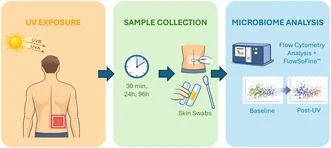

**Abbreviations**
cSCC: cutaneous squamous cell carcinoma
FC: flow cytometry
ICH: international Council for HArmonisation
iMED: individual minimal erythema dose
ITA: individual typolofy angle
NMDS: non‐metric multidimensional scaling
PERMANOVA: permutational multivariate analysis of variance
SCC: side scatter
UVA: ultraviolet A
UVB: ultraviolet B
UVR: ultraviolet radiation

## INTRODUCTION

As a barrier organ, the skin is in constant exchange with its environment and provides a variety of habitats with different ecological niches for a wide range of microorganisms and microbial communities [1], which contribute to maintaining skin health in many ways. In a reciprocal host–microbe interaction, the human skin microbiome plays a crucial role in immune regulation, pathogen defense, maintenance of cutaneous homeostasis and differentiation of the epidermis [2, 3]. Nowadays, it is generally accepted that an imbalance in this system can lead to chronic tissue inflammation and autoimmunity [4].

Recent studies have investigated how ultraviolet radiation (UVR)—one of the most pervasive environmental stressors—affects this vulnerable balance. While the effects of UVR on immunomodulation and carcinogenesis are well established [5, 6], its impact on skin microbial community structure remains underexplored. Experimental and clinical data suggest that UVR can induce both stimulatory and suppressive effects on microbial populations, leading to alteration of microbiota composition and potentially increasing the susceptibility to skin inflammation and dysbiosis. A recent review by Gilaberte et al. [7] summarizes the current evidence indicating solar radiation can disrupt skin microbiota homeostasis. The authors also propose novel approaches to photoprotection aimed at preserving both skin and microbial integrity. This appears particularly relevant given growing evidence that the development of actinic keratoses and cutaneous squamous cell carcinoma (cSCC)—both diseases primarily caused by chronic sun exposure—is associated with changes in the skin microbiota composition [8]. Furthermore, experimental data in mice suggest that UVB‐induced immunosuppressive effects can be modulated by the presence and composition of the skin microbiota and their metabolic products [9, 10]. This highlights the relevance of investigating UVR‐induced alterations in the microbiota not only from an ecological perspective, but also in the context of therapeutic and preventive applications in dermatology.

The primary objective of this study was to investigate short‐term and intermediate changes in the skin microbiota following controlled exposure to simulated solar radiation (UVB plus UVA).

To date, data on dose‐ and time‐dependent alterations in the skin microbiome following UV‐exposure remain scarce. This is largely due to the considerable time and expense associated with conventional sequencing‐based microbiome analyses, particularly when a large number of time points and exposure levels need to be assessed. In this context, flow cytometry (FC)‐based approaches offer a cost‐ and time‐efficient alternative [11]. Although lacking taxonomic resolution, FC‐based methods enable the detection of global shifts in microbial community structure and are therefore powerful screening tools to identify relevant exposure threshold and time points for more detailed downstream analyses. A recent study has demonstrated that FC‐based microbiota analysis can reliably differentiate between microbiota profiles of distinct skin sites [12]. However, this method has not yet been applied in a longitudinal study setting. Building on this previous work, the present study aims to establish FC as a longitudinal screening method to monitor dynamic changes in skin microbiota composition over time in response to defined UV exposures.

## MATERIALS AND METHODS

### Study design

This exploratory, monocentric *in* *vivo* study was conducted at the study center of the Leibniz Research Institute for Environmental Medicine in Duesseldorf (Germany) from January 31 to April 3, 2023. The study was approved by the Ethics Committee of the Medical Faculty of Heinrich Heine University (Ethic no. 2022‐2150) and conducted in accordance with the guidelines of the Declaration of Helsinki and the guidelines of the ICH (International Conference on Harmonization).

### Study subjects

Thirty healthy Caucasian males aged between 22 and 45 with intermediate skin pigmentation in accordance with Chardon et al. [13] (typology angle [ITA°] between 29.91° and 40.95° [average ITA°: 35.91 ± 3.26]) were included in the study. Subjects with clinical signs or a history of relevant pathologies, in particular signs of abnormal light sensitivity (photosensitivity), were not included in the study. Subjects were not allowed to have a history of inflammatory or malignant skin diseases. Subjects with special diets (e.g., vegan or vegetarian eating habits), who smoked, consumed excessive amounts of alcohol (>1 alcoholic drink/day) or illegal drugs were also not included in the study. Subjects who took systemic (except paracetamol) or topical medications up to 30 days prior to inclusion in the study (especially antibiotics, immunosuppressants, or photosensitizing medications), had skin damage in the study area, suffered from immunosuppressive diseases or received immunosuppressive/‐modulatory therapies were also excluded. Particular attention was paid to possible interactions between pre‐existing diseases and their treatment on the microbiome.

During the study period of approximately 2 weeks, the subjects were prohibited from using sun beds or sunbathing. The subjects had to refrain from taking nutritional supplements from the preliminary examination until the end of the study. The use of topical products (e.g., sunscreen) in the study area on the lower back was prohibited. In addition, the subjects were provided with a shower gel free from microplastic, nanoparticles, and with skin‐neutral pH, which had to be used exclusively for personal hygiene during the study. This was to prevent interference with the measurement methods.

### Study procedures

**FIGURE 1:**
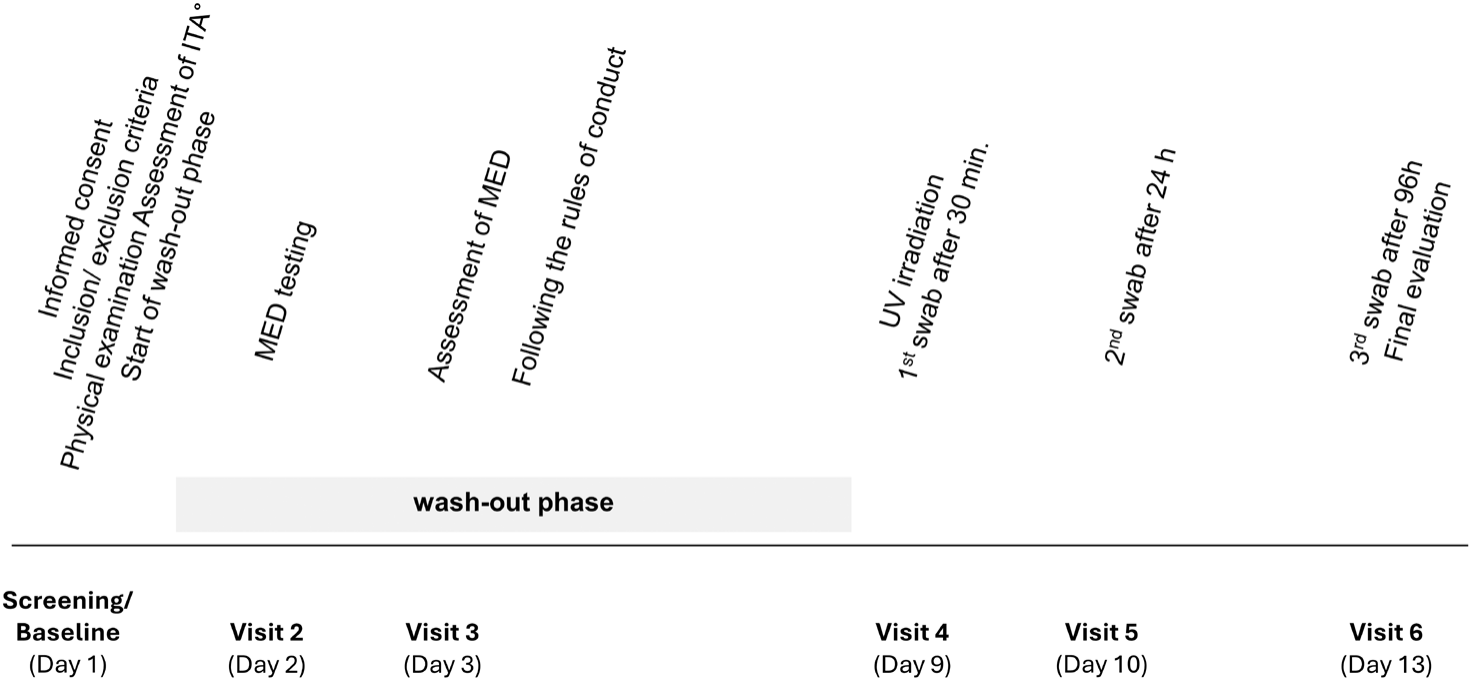
Study phases for each individual subject (MED, minimal erythema dose; ITA, individual typology angle).

All study procedures were carried out only after obtaining written informed consent from each subject. At the beginning of the study, the individual minimal erythema dose (iMED) was determined for each subject. A solar simulator (BG‐02, Opsytec Dr. Gröbel GmbH, Germany); equipped with UVB (G15 T8E from Sankyo Denki, Japan) and UVA (Actinic BL TL‐D 15 W lamps from Philips, The Netherlands) lamps was used for iMED determination and for the experimental UV exposure carried out during the study. The device enables homogeneous UVA/UVB irradiation without exerting any thermal effects on the skin. The iMED was determined on an area of the lower back that had not been previously exposed to the sun. The maximum dose was 160 mJ/cm^2^ (UVB alone) and was reduced in 25% decrements across the six test fields. Sixteen to 24 h after irradiation, the iMED was visually assessed by expert grading. The average iMED of the subjects was 58.00 ± 0.018 mJ/cm^2^, which is within the normal range for Caucasians with ‘intermediate’ skin type [14]. For the experimental irradiation, 12 test areas (each 4 × 5 cm) were marked on the lower back, arranged in three sets of four fields. Three fields of each set were exposed to 0.5, 0.7, or 1.0 MED plus a UVA dose corresponding to approximately 41 times of the respective UVB dose (UVB to UVA ratio 1:41) [15]. One field of each set served as a control field. Skin swabs were taken from the irradiated areas at three time points (30 min, 24 h, and 96 h) after irradiation and were processed within 4 h according to a standardized protocol (see Supplementary Materials). At the same time points, swabs were taken from non‐irradiated skin as controls. The study comprised a total of six visits. Adverse events, medication use and compliance were assessed at each visit. Each subject's participation in the study was considered complete once all procedures had been carried out in accordance with the protocol. The detailed procedures carried out during a visit can be seen in Figure 1.

### Flow cytometric measurement and bioinformatic analysis of the skin microbiota

Further sample preparation and staining of the bacteria with the DNA dye Midori Green was carried out according to the protocol of Koch [16] and Zimmermann [17], modified by Kupschus et al. [12] as described in Supplementary Materials. Using a DNA‐binding dye enables selective detection of microbial events by ensuring that only particles with nucleic acids are included in the analysis. In FC, individual particles in suspension are interrogated by a laser, generating optical signals that allow their differentiation.

FC measurements relied primarily on side scatter (SSC) to capture differences in cell size and structural complexity. Microbial subpopulations were discriminated based on SSC intensity and DNA signal characteristics, which form discrete clusters representing phenotypically distinct bacterial groups. Exemplary density plots resulting from cluster‐based discrimination are shown in Figure S1 in the Supplementary Material.

The analysis of the composition of the microbiota was based on all 354 samples from the 30 subjects. Incomplete sample sets per subject were also included in the statistical analysis. Three sample sets were incomplete (2 samples were lost due to technical issues during sample preparation; one subject missed visit 5).

The bioinformatic analysis and visualization of the composition of the microbiota were performed with the R package FlowSoFine™ (described in detail by Kupschus et al. [12]). Bray‐Curtis distances were calculated to quantify the differences between samples, and the results were visualized using nonmetric multidimensional scaling (NMDS). To account for the longitudinal structure of the dataset, group differences in the composition of the microbial community were tested using a modified PERMANOVA with a restricted permutation design for repeated measurements, implemented via the R‐Package *permute* (version 0.9‐7) (with how(plots = Plots(meta$ID, type = “none”), within = Within(type = “free”), nperm = 5000)) [18]. This approach takes into account the dependency of within‐subject measurements.

Results were considered to be statistically significant with *p*‐values < 0.05, and multiple testing correction was done using Holm's method [19].

To identify regions within the scatter plots that contribute most to group separation, bin‐wise scores were calculated using the Welch t‐statistic and projected as heatmaps onto the template. These so‐called t‐scores facilitated the visual localization of bacterial clusters responsible for the observed differences. Changes in cluster position or intensity represent quantitative shifts in the relative abundance of the corresponding microbial sub‐population (see Supplementary Material for details).

## RESULTS AND DISCUSSION

(Sub‐) erythemal exposure to solar‐simulated UVR (0.5, 0.7, 1.0 MED) induced measurable changes in the skin microbiota. In particular, significant alterations in the microbial composition were already observed approximately 30 min after irradiation across all three dose levels compared to the control sites. These immediate changes were present even at moderate suberythemal doses (0.5 and 0.7 MED), indicating that minimal UV exposure is sufficient to cause shifts in the skin microbiota.

**FIGURE 2:**
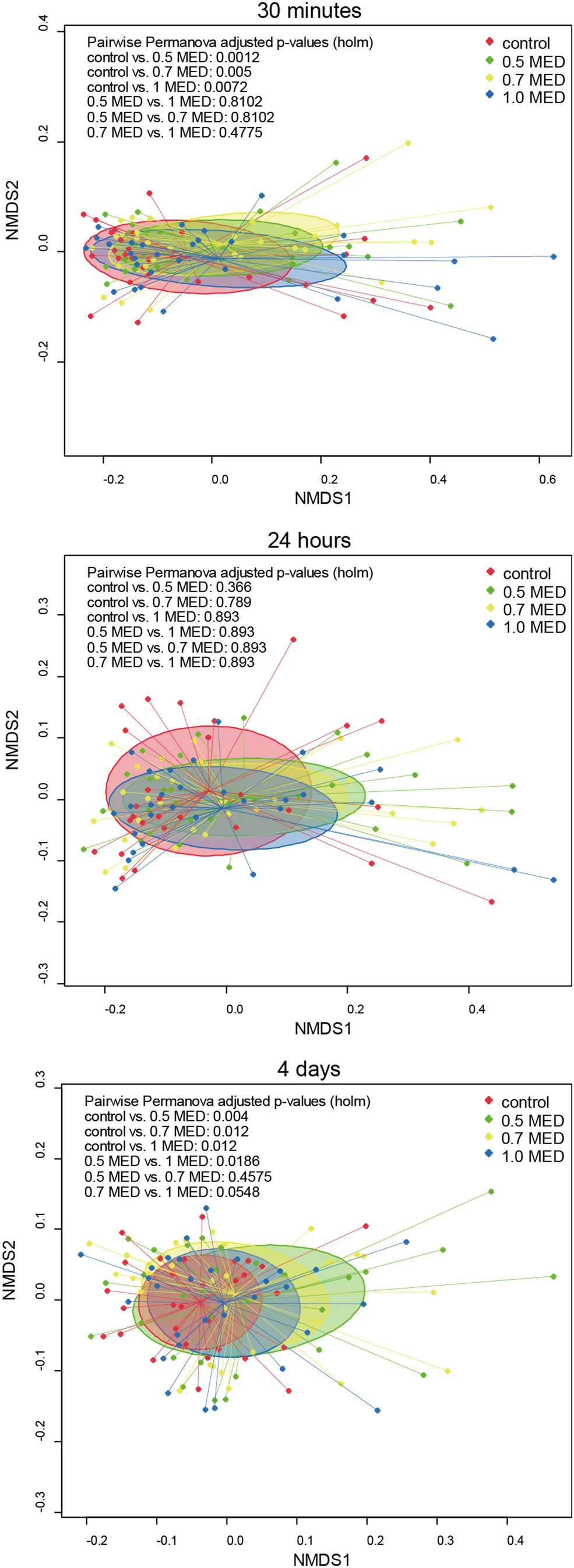
Nonmetric multidimensional scaling (NMDS) plots based on Bray‐Curtis distances illustrating differences in microbial community composition at each time point compared to time‐matched controls. Each point represents the community profile of a single sample derived from FlowSoFine™ cluster structure. Colored ellipses indicate the group centroid and its 95% confidence region. Overlap indicates similarity between groups. Greater distance between ellipses reflects larger compositional differences (MED, minimal erythema dose).

The resulting distances between the three time points within each dose level were visualized using NMDS plots (Figure 2).

**FIGURE 3:**
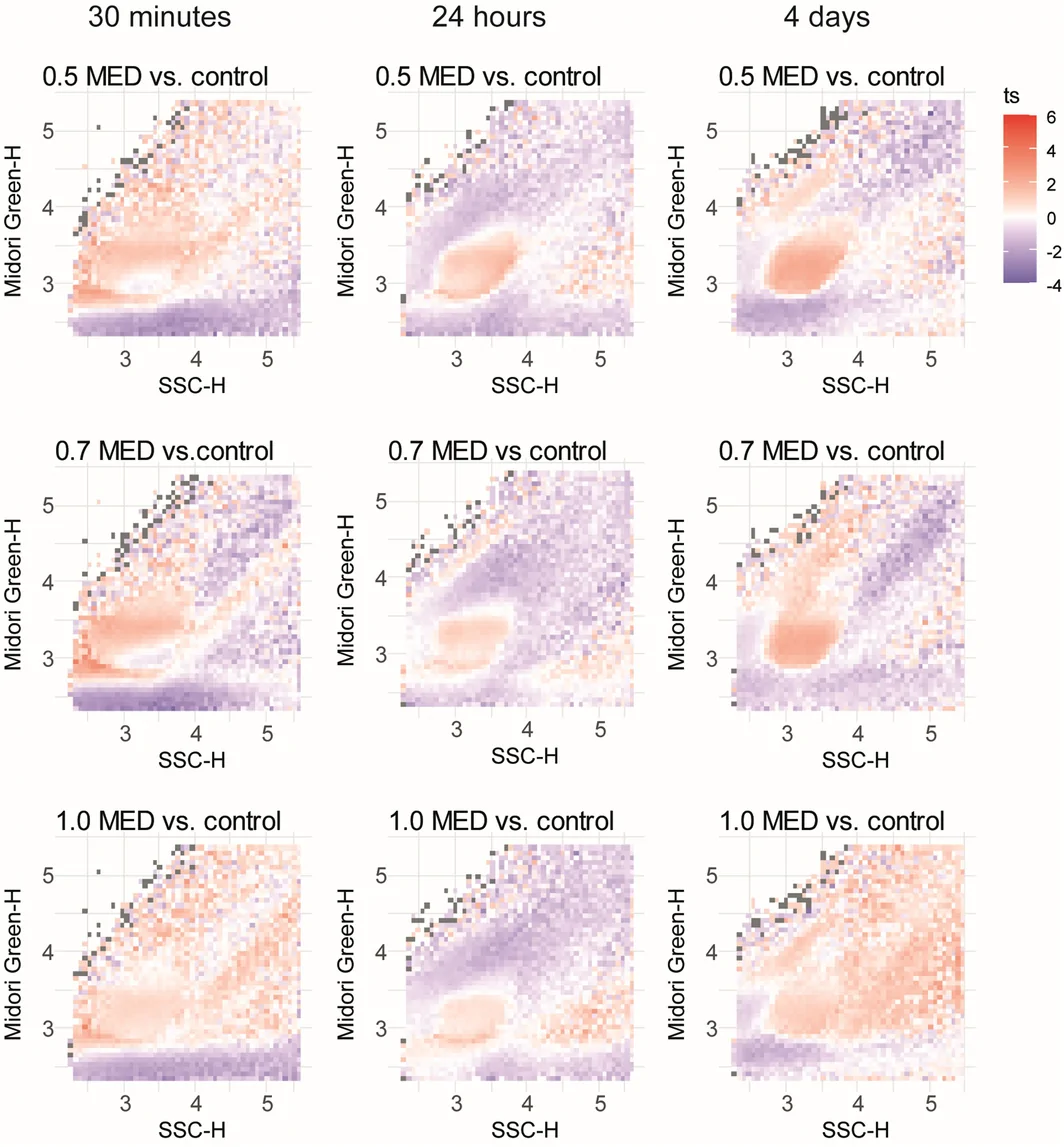
T‐scores at each time point (30 min, 24 h, 4 days) and UV dose (0.5, 0.7, 1.0 MED) relative to the respective time‐matched control sample. Blue color indicates a reduction and red color indicates an increase in bacteria compared to the control (MED, minimal erythema dose; Midori‐Green Height (DNA content); SSC‐H, side scatter height (~cell size)).

The application of the t‐score based visualization [12] and subsequent heat map analysis (Figure 3) revealed region specific differences in microbial alterations after UV exposure depending on the applied UV dose. After 4 days, pronounced differences in microbiota alterations are visually apparent, particularly between 1 MED and 0.5 MED irradiated fields. This observation is supported by statistically significant differences in microbiota composition between the respective irradiated areas (0.5 MED vs. 1.0 MED after 4 days, *p* = 0.019) (see Supplementary Material, Figure S3). These regions correspond to clusters of bacteria with shared optical and structural properties. This supports the hypothesis that the impact of UV radiation on the skin microbiome is dose‐dependent and may selectively affect microbial subpopulations based on their UV susceptibility.

At the 24‐h time point, the initial microbial shifts had largely subsided, and no statistically significant differences were detected compared to the nonirradiated control fields sampled at the same time points. However, by day 4, new or recurring changes became apparent, with significant differences between irradiated and time‐matched nonirradiated control fields. These findings could reflect delayed community‐level responses to UV‐induced stress.

Since the analysis of the microbiome over time showed instability in the microbial patterns in the control fields (Control: baseline vs. final visit; *p* = 0.032), the analysis of the long‐term effects (after 4 days) requires caution.

It cannot be ruled out that the observed instability represents natural microbiota variability or is a direct consequence of UV exposure. The control areas were located only few centimeters from irradiated fields, making lateral microbe migration from UV irradiated fields conceivable. In addition, controlling for individual confounding factors (e.g., lifestyle factors such as individual stress level or sleep quality, as well as mechanical irritation) over several days is inherently challenging. Spatial variation between sampling sites may also have contributed: baseline swabs were taken at the level of the midback, whereas samples on day 4 were collected further down the lower back. Thus, differences in the local skin microenvironment may have influenced the results. Ultimately, a definitive conclusion cannot be drawn.

Taken together, these factors limit the ability to attribute delayed changes exclusively to UV exposure. Future studies should employ more distant control sites, strictly identical sampling locations across all time points, and additional longitudinal sampling to better distinguish natural temporal variability from exposure‐driven effects.

**FIGURE 4:**
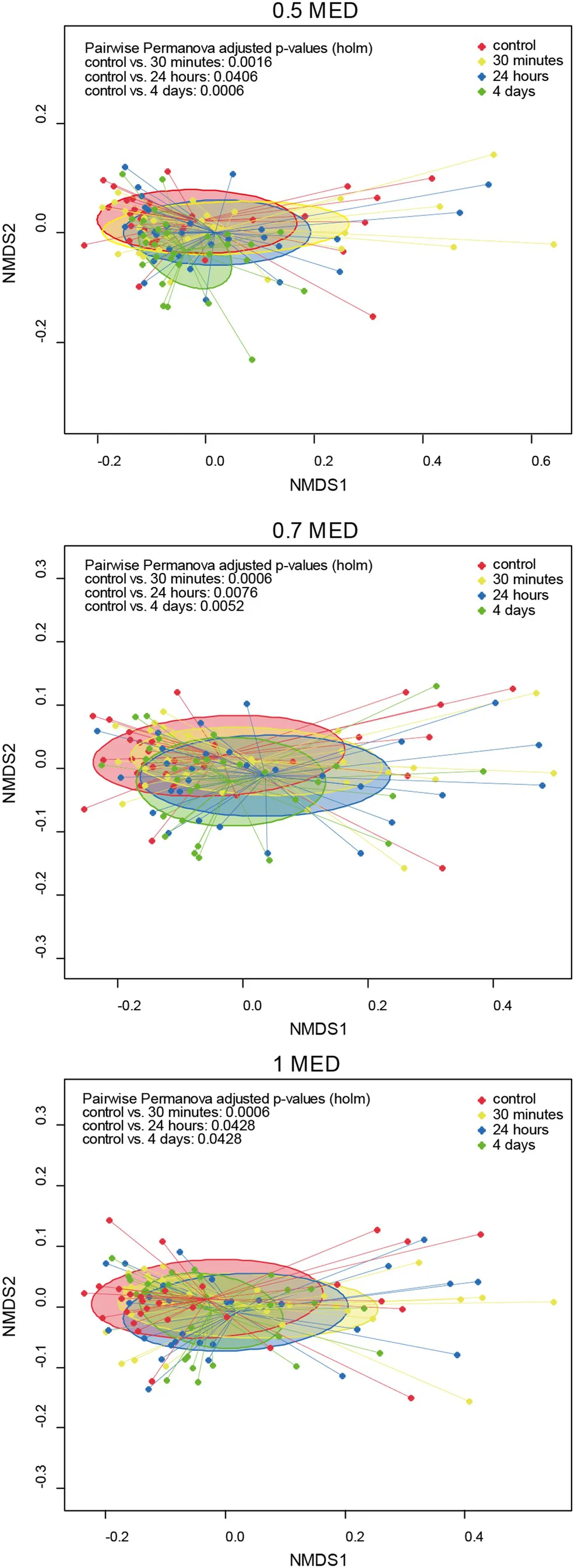
Nonmetric multidimensional scaling (NMDS) plot based on Bray‐Curtis distances illustrating differences in microbial community composition after UV exposure relative to baseline control samples (from nonirradiated skin sites 30 min after UV exposure). Each point represents the community profile of a single sample derived from FlowSoFine™ cluster structure. Colored ellipses indicate the group centroid and its 95% confidence region. An overlap indicates similarity between groups. Greater distance between ellipses reflects larger compositional differences (MED, minimal erythema dose).

Given the pronounced temporal alterations observed in the control fields, we performed a follow‐up analysis comparing the microbiome composition at each time point to the baseline composition within the control field (Figure 4).

This revealed significant differences in microbial composition at all‐time points and across all UV doses. Once again, the T‐score based visualization indicated dose‐dependent differences in microbiota changes at each time point, suggesting a potential association between the extent of microbial alteration and the level of UV exposure (see Supplementary Material, Figure S2).

To date, there is only limited knowledge about the short‐term (minutes) and intermediate (few days) effects of UV radiation on the skin microbiota. The first evidence of an influence of UV radiation on the skin microbiome was provided by a study from 2019 [20]. This study revealed that UV radiation had an effect on the skin microbiota that persisted up to 24 h post‐UV exposure. In general, an increase of the phylum Cyanobacteria was observed after both UVA and UVB irradiation. The authors attributed this to the evolutionary survival advantages of Cyanobacteria in UV‐exposed environments, in particular their ability to perform photosynthesis and their effective defense mechanisms against oxidative stress. In addition, a decline in Lactobacillaceae (phylum Bacillota) and Pseudomonadaceae (phylum Pseudomonadota) was observed at the family level. However, the applied UV doses (22–47 J/cm^2^ UVA; 100–350 mJ/cm^2^ UVB) were substantially higher than in the present study, and UVA and UVB were administered separately [20].

A field study from 2023 [21] demonstrated that real‐life sun exposure during holidays transiently altered the skin microbiome. Significant shifts—particularly a reduction in Proteobacteria—in the structure of the microbial community were observed 1 day after returning from sun‐intensive holidays in participants who had experienced visible tanning and reported sun‐seeking behavior. It is noteworthy that microbial diversity and taxonomic composition returned to baseline levels within 28 days of exposure, suggesting reversibility of sun‐induced effects.

In a seasonal comparative study [22], microbiome profiles of two populations with differing UV exposure levels were analyzed. Despite prolonged UV exposure in one group (lifeguards), the overall taxonomic structure of the skin microbiome remained remarkably stable at the phylum level. Only subtle but consistent changes in low abundant taxa were observed in individuals with high levels of UV exposure. These results suggest a resilience of the core microbiota to environmental UV exposure, while minor taxa may respond more susceptible and potentially drive UV‐associated skin responses.

Initial clinical data [23] indicate that topical sunscreen (SPF 20) can protect the skin microbiome from acute UV‐induced (2 MED) changes (2 h after exposure). Supplementary *in* *vitro* data provided evidence that selected UV filters may preserve the viability of key commensals such as *Lactobacillus crispatus*, *Cutibacterium acnes*, and *Staphylococcus epidermidis* [23].

The comparability of these studies is limited due to different exposure conditions, differences in microbiome analysis and, in some cases, due to the examination of different body sites. It is well known that the microbial composition varies considerably in different skin regions. The human back provides a sebum‐rich environment with low overall bacterial diversity [24]. The dominant taxa include *Cutibacterium* spp., which can metabolize free fatty acids, as well as *Staphylococcus aureus* and *Staphylococcus epidermidis* [3]. In the present study taxonomic sequencing was not performed. Therefore, microbial identity at the species level cannot be confirmed, nor can their functional relevance be interpreted. As a result, any discussion of specific taxa remains based on known ecological characteristics of this anatomical niche rather than on direct taxonomic profiling.

Several—primarily *in* *vitro*—studies have suggested that skin microbiota provide a wide range of molecular mechanisms of protection against UV‐induced damage [25], from enzymatic metabolism and antioxidant stress protection [26, 27, 28, 29, 30] to immunomodulatory effects [31]. Recent mechanistic data further support this concept. Patra et al. demonstrated that urocanase‐positive skin‐resident bacteria can metabolize UV‐induced cis‐urocanic acid and thereby reduce its immunosuppressive effects [10]. Moreover, Zarfl et al. showed that eradication of the skin microbiota restores cytokine production in polymorphic light eruption, highlighting the microbiome's contribution to UV‐associated immunomodulation [32]. These mechanisms open up promising avenues for microbiota‐based photoprotection strategies.

Further high‐quality clinical studies investigating the effects of UV radiation on the microbiota and the associated influence on microbiota‐host interaction, as well as the possible photoprotective effects of the microbiota itself, are highly desirable. In addition, further placebo‐controlled interventional studies are needed to investigate the possibilities of protecting the skin microbiome from UV radiation.

## LIMITATIONS

This exploratory study has several limitations. The cohort was small, included only men, and investigated only one anatomical site, which restricts generalizability. Day‐4 samples were collected at a slightly different skin location, introducing potential spatial variation. In addition, the close proximity of irradiated and control fields may have allowed lateral microbial migration. Natural temporal variability of the skin microbiota cannot be excluded, particularly regarding changes at later time points.

Microbiome profiling was performed using quantitative FC without taxonomic sequencing. Thus, species‐level resolution and mechanistic interpretations are not possible.

Future studies with larger and more diverse cohorts, stable longitudinal sampling at identical sites, and integrated taxonomic and functional profiling are required to confirm and extend these findings.

## CONCLUSION

This study demonstrates that even suberythemal doses of solar‐simulated UV radiation (UVB plus UVA) induce measurable and partially reversible shifts in the composition of the human skin microbiota. Given that the general population is regularly exposed to suberythemal UV doses during daily life, these findings are likely to be of clinical relevance.

Our study supports and expands limited existing clinical data that UV radiation is a disruptor of the skin microbial ecosystem. The functional consequences of UV‐induced microbiome disruption, including its implications for skin health and disease susceptibility, remain unclear and warrant further investigation. In this context, the potential protective effects of topical agents such as sunscreens should be systemically investigated.

Furthermore, FC‐based microbiota profiling emerges as a robust, cost‐effective screening approach to detect community level changes and to identify UV exposure levels and time points associated with most pronounced microbiota shifts. This approach can enable more targeted downstream microbiome analyses, including 16S rRNA or shotgun metagenomics sequencing, which can optimize resource allocation in longitudinal or dose–response studies.

## AUTHOR CONTRIBUTIONS

CE, FD, CT, AB, MF, and JK designed the study. FD, DR, and CW conducted the analysis and data interpretation. DR created the illustrations. FD and JK performed the study. FD, JK, and JPC contributed to the original draft. All authors edited, reviewed, and approved the final version.

## FUNDING INFORMATION

This study was funded by ISDIN, Barcelona, Spain.

## CONFLICT OF INTEREST STATEMENT

JPC is a consultant for ISDIN. CT, AB, and MF are employed by ISDIN, which sponsored the study. JK is the Director of IUF and receives fees from ISDIN. No other conflicts of interest were declared.

## Supporting information


Data S1:

## Data Availability

The data that support the findings of this study are available from the corresponding author upon reasonable request.
