# Supplementary material for: Effects of solar‐simulated (UVB plus UVA) radiation on the skin microbiome: An exploratory study
Source: Photochem Photobiol. 2026 Jan 29;102(5):1150–8. doi: 10.1111/php.70078 (PMC13575714; doi:10.1111/php.70078)
Supplement: Supplementary file 1 [file PHP-102-1150-s001.docx]

SUPPLEMENTARY MATERIALS

**Sample collection and preparation**

Microbiota samples were collected from designated 4 x 5 cm skin areas using sterile cotton swabs premoistened with isotonic and sterile saline. The swabs were wiped with light pressure over the skin. The sample was then transferred to sterile Falcon tubes containing saline solution by shaking the swab vigorously several times in the tube. Samples were stored at 4 °C and processed within four hours. First, the samples were filtered through a sterile sieve with a mesh size of approximately 100 µm to remove any contamination. Bacterial cells were pelleted by centrifugation (3200 x g, 5 min, 4°C), fixed in 500 µl of 2% formaldehyde diluted in PBS for 30 minutes at room temperature, washed with cold phosphate-buffered saline (PBS), resuspended in 70% ethanol, and stored at -20°C.

Prior to staining, fixed microbes were washed with sterile filtered PBS and incubated in a permeabilization puffer (0.11 M citric acid, 4.1 mM Tween®20) for 20 minutes at room temperature. The microbiota was then centrifuged (3200 x g, 10 min, 14°C) and resuspended in staining buffer (pH 7, 400 mM sodium phosphate) containing the DNA-dye Midori Green Advance (1:50,000 dilution; Nippon Genetics). Staining was carried out for 1 hour at room temperature under light-protected conditions.

Flow cytometry measurements were performed using a FACSAria™ III (BD Biosciences, San Jose, CA, USA) equipped with a 488 nm laser (> 20mW), with FACSDiva™ Software Version 6.1.3. Instrument performance was validated prior to each run using MEGAMIX Plus SSC beads (Diagnostica Stago, Asnières sur Seine, France).

To distinguish bacterial populations, fluorescence (Midori Green-H) and the side scatter (SSC-H) signals were recorded using photomultiplier tubes with bandpass filters of 530/30 nm and 488/10 nm, respectively. Given the small size of bacterial cells, SSC-H was used in place of forward scatter (FSC) to improve detection sensitivity. To minimise background noise, the lowest technically possible threshold value of 200 was applied to both channels. A total of 110,000 events per sample were recorded on a logarithmic scale across all 354 samples. Sterile, filtered PBS was used as sheath fluid.

**Figure S1.** **Exemplary density plots generated by flow cytometry of one participant.** The y-axis shows Midori-Green fluorescence intensity (DNA content), and the x-axis shows side-scatter signal (SSC-H), reflecting approximate cell size/ structural complexity. Data were acquired using a BD FACSAria™ III. The smallest technical possible threshold value of 200 was used for both channels to eliminate background noise. Distinct density regions indicate flow-cytometrically differentiated microbial sub-populations and serve as the basis for subsequent analyses using FlowSoFine™. Colour indicates event density on a log10 scale, ranging from blue/green (low density) to yellow/ red (high density).

**Bioinformatic analysis**

The bioinformatic analysis was performed using the R package FlowSoFine™, as described in detail in Kupschus et al. (1). The analyses were performed using the R statistical programming language (version 4.3.0) (2). FCS (Flow cytometry Standard) files were imported using the flowCore package (3) and visualizations were generated using the ggplot2 package (4). Raw data were log-transformed and subsequently discretized using the data.table package (5) and dense_rank function from the dplyr package (6).

To determine an appropriate resolution for multidimensional binning, the Normalized Sparsity Reduction (NSR) method was applied, resulting in 20 bins per axis. After excluding bins without any events across all samples, this yielded approximately 338 bins per sample.

After import, all FCS files were merged into a single dataset. A grid template was then created and all individual scatter plots were aligned to the grid, resulting in a dataset that represented the proportion of events per bin for each sample.

Bray-Curtis dissimilarities were calculated using the discretized and normalized (0 to 1) data to assess the between-samples distances. The results were visualised via non-metric multidimensional scaling (NMDS).

Group differences were assessed using a modified Permuted multivariate analysis of variance (PERMANOVA) (7) with 5,000 permutations, performed via the ‘adonis2’ function from the ‘vegan’ package, with a fixed random seed (131) to ensure reproducibility (8).

Given the longitudinal study design with repeated-measurements for each participant, we employed a restricted permutation scheme using the ‘how( )’ function from the ‘permute’ package in R (9). Permutations were only allowed within each subject, randomizing the assignment of timepoints and areas. No permutations were performed across subjects (type = “none”). This approach preserves the intra-subject structure of the data, avoids redundant subject-level permutations (which do not contribute to the group comparison), and enables inclusion of subjects with incomplete measurements.

The r code is extended as follows:

how(plots=Plots(meta$ID, type="none"), within = Within(type = "free"), nperm=5000)

This configuration tolerates a certain imbalance in the number of samples per subject, allowing incomplete data sets to be included in the analysis. However, significant imbalance combined with heterogeneous group distribution should be avoided, as this could compromise the validity of the results (10). Results were considered statistically significant with p-values ≤ 0.05, and multiple testing correction was done using Holm´s method (11).

Furthermore, the key discriminatory features were identified using the t‑score approach introduced by Kupschus et al. (1). Bin-wise scores were calculated based on Welch t-statistic and projected onto the template as heatmaps. These t-scores enable the spatial localisation of bacterial clusters responsible for the observed group differences.

**Figure S2. T-scores at each time point (30 min, 24h, 4 days) and UV dose (0.5, 0.7, 1.0 MED) relative to baseline control samples.** Blue colour indicates a reduction and red colour indicates an increase in bacteria compared to the control (MED: Minimal Erythema Dose; Midori-Green Height (DNA content); SSC-H: Side Scatter Height (~cell size)).

**Figure S3. T-scores at day 4 comparing the microbial community fingerprints between 0.5 MED and 1.0 MED UV exposure.** Blue colour indicates a reduction and red colour indicates an increase in bacteria after exposure to 1.0 MED compared to 0.5 MED (MED: Minimal Erythema Dose; Midori-Green Height (DNA content); SSC-H: Side Scatter Height (~cell size)).

**References**

1. Kupschus, J., S. Janssen, A. Hoek, J. Kuska, J. Rathjens, C. Sonntag, K. Ickstadt, L. Budzinski, H. D. Chang, A. Rossi, C. Esser and K. Hochrath (2023) Rapid detection and online analysis of microbial changes through flow cytometry. *Cytometry A* **103,** 419-428.

2. R Core Team (2023) R: A language and environment for statistical computing. R Foundation for Statistical Computing, Vienna, Austria.

3. Ellis, B. et al. (2023) flowCore: Basic structures for flow cytometry data. R package version 2.0.1.

4. Wickham, H. (2016) ggplot2: Elegant graphics for data analysis. Springer, New York.

5. Dowle, M. and A. Srinivasan (2021) data.table: Extension of ‘data.frame’. R package version 1.14.0.

6. Wickham, H., R. François, L. Henry and K. Müller (2021) dplyr: A grammar of data manipulation. R package version 1.0.7.

7. Anderson, M. (2001) A new method for non-parametric multivariate analysis of variance. *Austral Ecology* **26,** 32-46.

8. Oksanen, J. et al. (2020) vegan: Community Ecology Package. R package version 2.5-7.

9. Simpson, G. L. (2023) permute: Functions for generating restricted permutations of data. R package version 0.9-7.

10. Anderson, M. J. and D. C. I. Walsh (2013) PERMANOVA, ANOSIM, and the Mantel test in the face of heterogeneous dispersions: What null hypothesis are you testing? *Ecological Monographs* **83,** 557-574.

11. Holm, S. (1979) A Simple Sequentially Rejective Multiple Test Procedure. *Scandinavian Journal of Statistics* **6,** 65-70.
